# Supplementary material for: Presence of antiphospholipid antibodies is associated with increased implantation failure following in vitro fertilization technique and embryo transfer: A systematic review and meta-analysis
Source: PLoS One. 2022 Jul 27;17(7):e0260759. doi: 10.1371/journal.pone.0260759 (PMC9328555; doi:10.1371/journal.pone.0260759)
Supplement: S1 Appendix — (DOCX) [file pone.0260759.s001.docx]

**Appendix 1. Search Strategies**

**Medline/Cochrane library**

#1 Antiphospholipid syndrome [mh]

#2 Antibodies, antiphospholipid [mh]

## Antiphospholipid

#3 Phospholipids/immunology[mh]

#4 Antiphospholipid [tiab]

#5 Anti-phospholipid[tiab]

#6 anticardiolipin [tiab]

#7 anti-beta2 glycoprotein I[tiab]

#8 lupus anticoagulant[tiab]

#9 phosphatidylserine[tiab]

#10 phosphatidylcholine[tiab]

#11 phosphatidylethanolamin[tiab]

#12 phosphatidylinositol [tiab]

#13 phosphatidyl-glycerol [tiab]

#14 phosphatidic acid [tiab]

#15 /OR 1-14

#16 Infertility, Female [mh]

#17 Infertility, Male [MH] NOT (Infertility, Female [MH])

#18 Infertility [mh] not #8

#19 Fertility Preservation [MH]

#20 Fertility Agents, Female [mh]

#21 Fecundity[mh]

#22 Fecundability [mh]

#23 Ovarian Reserve [mh]

#24 infertility [tiab]

#25 ovarian reserve [tiab]

## Fertility

#26 conception [tiab]

#27 endometrium [tiab]

#28 implantation [tiab]

#29 endometrial implantation [tiab]

#30 /OR 16, 18-29

#31 male [mh] not (female[mh])

#32 #30 not #31

#33 #15 and #32

#34 animals[mh] not humans [mh]

#35 #33 not #34

**Appendix 2. Characteristics of the included studies**

**Coulam 1997**

Reference: Carolyn B. Coulam, Brian D. Kaider, Azadeth S. Kaider, Patrick Janowicx, and Roumen G. Roussev. Antiphospholipid Antibodies Associated with Implantation

Failure After IVF/ET. Journal of Assisted Reproduction and Genetics, Vol. 14, No. 10, 1997

| **Methods** | Group of 312 women with implantation failure was compared with group of 100 fertile control women. An implantation failure was defined as no signs of implantation after transfer of 12 or more embryos or the occurrence of two or more pregnancy losses during previous transfer attempts. All women had unexplained IVF/ET failure without obvious ovulatory, male, or uterine factors. |
| --- | --- |
| **Participants** | To be included in the implantation failure group, each woman had to have had at least 12 embryos transferred without subsequent positive pregnancy test. The number of years of infertility, the cause of infertility and the number of previous clinical, ectopic, and biochemical pregnancies were recorded. The numbers of oocytes retrieved and fertilized, and of embryos  transferred during previous IVF/ET attempts, were noted. |
| **Interventions** | ELISA assay was used to measure IgG, IgM, and IgA anticardiolipin, antiphosphatidyl ethanolamine, antiphosphatidyl inositol, antiphospatidic acid, antiphosphatidyl glycerol, antiphosphatidyl choline and antiphosphatidyl serine. The reaction was stopped with 3 *M* NaOH, and the color optical density was detected with a Bio-Kinetics EL312e  ELISA reader. |
| **Outcomes** | Positive antiphospholipid antibodies (aPL) were detected in 69 (22%) of the 312 women with implantation failure compared with 5 (5%) of the 100 control women (P < 0.0001). Anticardiolipin antibodies were found in 13 (4%) of the 312 women with implantation failure and none of the controls. Fifty-six (18%) of the 312 with implantation failure were negative for anticardiolipin antibodies but had positive values of other aPL. |

**Vaquero 2006**

Elena Vaquero, Natalia Lazzarin, Donatella Caserta,Herbert Valensise, Marina Baldi, Massimo Moscarini, Domenico Arduini. Diagnostic evaluation of women experiencing repeated in vitro fertilization failure. European Journal of Obstetrics & Gynecology and Reproductive Biology 125 (2006) 79–84

| **Methods** | Group of 59 patients with at least two unsuccessful IVF attempts were included in the study and compared with 20 healthy controls. |
| --- | --- |
| **Participants** | In the period of January 2003 to December 2003 59 non-pregnant patients with at least two unsuccessful IVF attempts were included in the study. These past IVF attempts were characterized by good quality embryos (i.e. regular blastomeres and no minor fragments). This group compared with 20 non pregnant healthy fertile women had at least two previous ncomplicated pregnancies. No differences were observed between groups in term of age and BMI. |
| **Interventions** | ELISA assay (Coaliza, Chromogenix. Moelndal, Sweden) enzyme-linked immunoabsorbent assays was used to measure anticardiolipin antibodies and β-2-glycoprotein IgM and IgG class. Test was considered positive when anticardiolipin antibodies of the IgG and/or IgM isotype, at  the medium (20–40) or high titer (>40) where present in  blood on two or more occasions at least 6 weeks apart. |
| **Outcomes** | The presence of anti-phospholipid antibodies (aPL) was detected in 19% (11 out of 59) of women experiencing IVF failure, whereas none of the healthy patients were aPL positive. Among the 11 positive patients, 6 were positive for aPL, 3 were positive for lupus anticoagulant and 2 for both. |

**Alves 2005**

J Delgado Alves, EL Radway-Bright, S Lee, B Grima, J Hothersall, CT Ravirajan, DA Isenberg. Antiphospholipid antibodies are induced by in vitro fertilization and correlate with paraoxonase activity and total antioxidant capacity of plasma in infertile women. Lupus,2005;14(5):373-80

| **Methods** | Group of 70 infertile women (18 before IVF, 13 submitted to one IVF cycle and 39 after three cycles) compared with 28 healthy controls. |
| --- | --- |
| **Participants** | From 70 infertile females undergoing routine IVF treatment, 52 patients were divided into two groups: I🡪after one IVF cycle (n=13) and II🡪after three IVF cycles (n=39), a group of infertile women before the first IVF treatment (n=18) was included as a second control group. *(This group will not be included in the meta-analysis)*.Of those who had three IVF cycles, 11 had a successful outcome after the third cycle and 28 were unsuccessful (18 miscarriages and 10 implantation failures). 28 age-matched healthy fertile women with at least one healthy pregnancy selected as control group. |
| **Interventions** | ELISA assay was used to measure anticardiolipin (Polysorp,  Nunc, Life Technologies, Paisley, UK) and antiphosphatidylserine (Sigma, Poole, Dorset, UK) IgG and IgM. Statistical analysis was performed using the Statistical Package for Social Sciences (SPSS). Nonparametric tests were employed to compare differences between groups (Kruskall–Wallis test) and to evaluate associations between variables (Spearman’s rank) Values >3 standard deviations  above the mean of fifty healthy controls were deemed to  be positive. |
| **Outcomes** | 56% of the 52 patients undergoing IVF treatment were positive for IgG aCL and 96% were positive for IgM aCL, 92 % were positive for anti-PS IgG and 27% for anti-PS IgM. None of the fertile women used as  normal controls or the infertile patients used as second control group were positive for aCL or anti-PS antibodies. There was a significant difference between antiphospholipid titers, when the IVF patients were  compared with infertile patients and normal controls (P <0.001 for both isotypes of aCL and anti-PS). |
| **Notes** | The 52 patients who had received treatment were also divided into four groups according to fertility diagnosis: male partner (n = 19), tubal (n =21), endometriosis (n =4) and unexplained infertility (n= 8).one of the patients had any other disease. There were no significant differences in aCL and anti-PS titers with different types of infertility. |

**Ulcova-Gallova 2005**

Zdenka Ulcova-Gallova, Vladimir Krauz, Pavla Novakova, Lucie Milichovska, Zdenka Micanova, Katerina Bibkova, Renata Sucha, Jiri Turek, Miroslav Balvin, Zdenek Rokyta. Anti-Phospholipid Antibodies against Phosphatidylinositol, and

phosphatidylserine are More Significant in Reproductive Failure than Antibodies against Cardiolipin only. American Journal of Reproductive Immunology 54 (2005) 112–117

| **Methods** | 2965 women with reproductive failure compared with 391 healthy fertile women. |
| --- | --- |
| **Participants** | A total of 2965 patients aged 22–44 years (average 27.5 years) attended the Special Division for Infertility and Reproductive Immunology at the Department of Obstetrics and Gynecology, Charles University, and Faculty Hospital, Pilsen, Czech Republic during 1998–2003.  Group 1=1073 women after one in vitro fertilization (IVF), group 2=853 women after two and more IVF, group 3= 627 women after three and more repeated spontaneous miscarriages or missed abortions, group 4= 412 women after diagnostic laparoscopy. *Groups 3 and 4 will not be includes in the meta-analysis.* |
| **Interventions** | ELISA assay was used for detection of anti-phospholipid against phosphatidic acid, phosphatidylethanolamine, phosphatidylinositol,  phosphatidylserine, phosphatidylglycerol (Sigma, St Louis, MO, USA), for anti- beta2-glycoprotein I (Immunotech, Prague Division, Czech Republic)  and anti-cardiolipin antibodies (Milenia, London, UK). |
| **Outcomes** | 928/1926 patients were positive for antiphospholipid antibodies(aPL), 421/1926 were positive for anticadiolipin antibodies, 778/1926 were positive for anti-phosphatidolserine and 209/1926 were positive for anti-β-2GLP1. 2/391 of the control group were positive for anti-phosphatylethanolamine in IgG (0.5%), 4/391for phosphatidylglycerol in IgM (1.0%), 5/391 for anti-caridolipin in IgG (1.3%) and 3/391 in IgM (0.77%). |

**Coulam 2002**

Coulam CB, Roussev R. Chemical pregnancies: immunologic and ultrasonographic studies. AJRI 2002; 48:323–328

| **Methods** | 122 women with implantation failure after IVF/ET versus 107 normal control women. |
| --- | --- |
| **Participants** | Group (1) of 122 women with implantation failure after IVF/ET associated with a negative pregnancy test, group (2) of 302 women experiencing two or more recurrent spontaneous abortions (positive controls), group (3) 107 normal control women (negative controls) all compared with group of 20 women who underwent IVF-ET and experienced a chemical pregnancy. A chemical pregnancy was defined as at least two rising values of serum human chorionic gonadotropin (hCG) concentrations without demonstration of a gestational sac after 3 week from ET. IVF implantation failure was defined as failure of implantation after transfer of cumulatively at least eight ‘good’ cleaving embryos or four blastocysts. |
| **Interventions** | ELISA assay was used to measure antiphospholipid antibodies (Sigma, St Louis, MO, USA). |
| **Outcomes** | Women experiencing chemical pregnancies had a higher frequency of aPL than fertile control women (16/20=80% versus 7/105=6%, P < 0.0001) and  women with implantation failure associated with a negative pregnancy test (16/20=80% versus 34/122=28%, P < 0.0001) and women experiencing  recurrent spontaneous abortion (16/20=80% versus 68/302=22%,  (p < 0.0001) |

**Sanmarco 2007**

M. SANMARCO, N. BARDIN, L. CAMOIN, A. BEZIANE,F. DIGNAT-GEORGE,M. GAMERRE, G. PORCU. Antigenic Profile, Prevalence, and Clinical Significance of

Antiphospholipid Antibodies in Women Referred for in Vitro Fertilization. Ann. N.Y. Acad. Sci. 1108: 457–465 (2007)

| **Methods** | 101 infertile women with at least three unsuccessful IVF attempts were consecutively included in this study and compared with age-matched healthy fertile women (n=160) were included as controls. |
| --- | --- |
| **Participants** | 101 non-pregnant women were recruited to this study between January 2003 and January 2000, referred for IVF treatment to three Centers for assisted Medical Procreations and Medical Institutes of Reproductive Medicine in the South of France. The inclusion criteria were age from 18 to 38 years (mean±SD, years=32±3.8) and at least two previous unsuccessful IVF-ETs.  Women with a history of prior pregnancy, one or more prior miscarriages, uterine malformation; a myoma; a history of clinical thrombosis or autoimmune disease; diabetes mellitus; a history of acute or chronic infectious disease; epilepsy or neuroleptic treatment were excluded.  The control population consisted of 160 non-pregnant, age-matched healthy  fertile women without any history of either reproductive problems or thrombosis or any autoimmune disorder or infectious disease. |
| **Interventions** | ELISA assay was used to measure anti-cardiolipin (aCL), anti-b-2-GPI,anti-phosphatidylethalamine (anti-PE) antibodies. The results were expressed in GPLU and MPLU for IgG and IgM-aCL, in arbitrary units for IgG and IgM-a_2GPI or aPE; in delta optical density for IgA isotype of aCL, a_2GPI, or aPE. The cut-off values were the following: 1/aCL-ELISA: IgG = 20 GPLU; IgM = 8 MPLU; IgA = 0.25; 2/a_2GPI-ELISA: IgG = 10 B2GU; IgM = 13 B2MU;IgA = 0.20;3/aPE: IgG = 15 PEGU; IgM = 54 PEMU; IgA = 0.15.  LA was performed by the kaolin cephalin clotting time utilizing sensitive reagents and by the dilute Russell’s viper venom time with a neutralization procedure using frozen–thawed platelets. Patients with an initial positive result for any aPL underwent a second test (12 weeks apart) just prior to the next ovulation induction treatment. The chi-square test with Fisher’s exact test was used to assess the relationship between antibodies and infertility or implantation. |
| **Outcomes** | 40/101 (39.6%) infertile women were persistently positive for at least one aPL whereas 8 controls were positive (8/160; *P <* 0.0001). 6/101 infertile women were positive for aCL, 24/101 infertile women were positive for anti-PE, 16/101 infertile women were positive for anti-β2GPI. No results for aCL , anti-PE, anti-β2GPI for control group are available. |

**Steinvil 2010**

Arie Steinvil, Raanan Raz, Shlomo Berliner, David M. Steinberg, David Zeltser, David Levran4, Orit Shimron; Tal Sella, Gabriel Chodick, Varda Shalev, Ophira Salomon. Association of common thrombophilias and antiphospholipid antibodies with success rate of in vitro fertilization. Thromb Haemost 2012; 108: 1192–1197

| **Methods** | 509 women with unexplained IVF failure were compared with 637 healthy controls. |
| --- | --- |
| **Participants** | Women with unexplained infertility initiating IVF treatments not older than 38 years from registry database of Maccabi Healthcare Services (MHS), undergoing IVF treatments from January 2000 through December 2010. Women with previous venous thromboembolism and/or treated  with low-molecular-heparin, as well as identified reason for infertility,  i.e. ovarian insufficiency, male factor, mechanical factors etc were excluded. |
| **Interventions** | ELISA assay (Orgentec, Mainz ,Germany) was used to measure immunoglobulin G anticardiolipin and beta 2 glycoprotein I antibodies. Lupus anticoagulant was determined by using a dilute Russell's viper venom time-based assay. |
| **Outcomes** | 17/509 of the study population were positive for at least one aPL whereas 30/637 of the control group were positive. |

**Paulmyer-Lacroix 2014**

Odile Paulmyer-Lacroix,Laura Despierres, Blandine Courbiere, Nathalie Bardin, Antiphospholipid Antibodies in Women Undergoing In Vitro Fertilization Treatment: Clinical Value of IgA Anti-𝛽2glycoprotein I Antibodies Determination. Hindawi Publishing Corporation BioMed Research International Volume 2014, Article ID 314704

| **Methods** | 40 women after at least two IVF failures compared with 100 healthy controls. |
| --- | --- |
| **Participants** | Women undergoing IVF from 2005 to 2011and no pregnancy occurred after at least two IVF attempts with good quality embryos available for the transfer (even cleavage, even cell sizes, <20% fragmentation). IVF treatment was performed at the Reproductive Department of University Hospital La Conception and was proposed to couples in female (ovulation disorders, tubal obstruction, and endometriosis), male, mixed, or unexplained infertility. Mean women’s age was 35 ± 4.15 years at the time of aPL detection. IVF indications were distributed as follows: female infertility (8 patients), male infertility (21 patients), mixed infertility (10 patients) and unexplained infertility (1 patient). |
| **Interventions** | ELISA Orgentec assay was used to measure anti-cardiolipin (IgM, IgG, and IgA) and anti-𝛽2GPI IgA antibodies.  For each aPL-ELISA, the cutoff level was determined by the analysis of the samples of 100 blood donors (control group) and was calculated at the 99th percentile. The results were expressed in GPLU and MPLU for IgG and IgM-aCL and a𝛽2GPI, in delta optical density, for IgA isotype of aCL  and a𝛽2GPI. The cutoff values were the following: 1/aCLELISA  : IgG = 20 GPLU; IgM = 8 MPLU; IgA = 0.25; 2/a2GPI-ELISA : IgG = 8 B2GU; IgM = 8 B2MU; IgA = 0.26 (GPLU, MPLU, and B2GU are arbitrary unit for, respectively, IgGIgMphospholipids and IgG𝛽2glycoprotein I antibodies). |
| **Outcomes** | Total prevalence of 20% (8/40) of aPL was found in study population significantly different from that of the control population (p < 0.0005)  Anti-𝛽2GPI IgA antibodies significantly higher in IVF patients (12.5%, 5/40) than in controls (1%, 1/100) (𝑃 = 0.01). |

**Khizroeva J 2018**

Jamilya Khizroeva, Alexander Makatsariya, Viktoriya Bitsadze, Natalya

Makatsariya , Nadin Khamani. In vitro fertilization outcomes in women with antiphospholipid antibodies circulation. The Journal of Maternal-Fetal & Neonatal Medicine;

| **Methods** | 178 consecutive women with previously failed IVF–embryo transfer (I group), 89 women with pregnancy after the IVF program (II group), compared with 80 pregnant women after IVF (male factor)(comparison group) and 80 pregnant women with physiological pregnancy (control group). |
| --- | --- |
| **Participants** | The study group comprised of 178 consecutive women with previously failed IVF–embryo transfer (I group), 89 women with pregnancy after the IVF program (II group). The comparison group consisted of 80 pregnant women after IVF (male factor). Control group included 80 pregnant women with physiological pregnancy. The age of patients ranged from 23 to 45 years old. The vast majority of women surveyed were multiparous with the absence of pregnancy for 3 to 17 years, provided regular sexual life without contraception. Clinical examination included data from medical histories, laboratory and instrumental investigations. The study excluded women with  chromosomal aberrations, anatomical defects and severe endocrine disorders. |
| **Interventions** | Fresh frozen plasma for homocysteine level (ELISA, ANТОS 2020, reagents Axis®, Axis-Shield AS, Norway), antiphospholipid antibodies (anticardiolipins, anti-b2-glycoprotein I, anti-prothrombin, antiannexin  V) (ELISA; Stago, Asserachrom APA) was used.  Evaluation of different hemostasis system associated genetic mutations and polymorphisms was performed by PCR analysis from fresh of frozen blood samples. The study was approved by the local Inter-University Ethics Committee of the Association of Medical and Pharmaceutical Universities. |
| **Outcomes** | aPL (total) were found positive in 42.1% in I group, 19.1% in II group, in 6.3% in comparison group and in 3.4% in control group.  Antibodies to cardiolipin IgG/ M were found positive in 8.9% in I group, 3.4% in II group, in 0% in comparison group and in 1.3% in control group.  LA were found positive in 19.6% in I group, 4.5% in II group, in 2.5% in comparison group and in 1.3% in control group.  Antibodies to b2GPI IgG/M/A were found positive in 31.4% in I group, 14.6% in II group, in 5% in comparison group and in 3.8% in control group. |

**Stern 1998**

Catharyn Stern, Lawrence Chamley, Lyndon Hale, Michael Kloss, Andrew Speirs, H. W. Gordon Baker. FERTILITY AND STERILITYtVOL. 70, NO. 5, NOVEMBER 1998

| **Methods** | 105 patients undergoing IVF without any resulting clinical pregnancy compared with fertile control group of 106 women. |
| --- | --- |
| **Participants** | 105 patients aged 24 to 47 years (mean age, 35 years) who enrolled in IVF program between January 6, 1996, and July 8, 1997 and who had undergone multiple ETs and previously had at least 10 embryos transferred without clinical pregnancy.  (A clinical pregnancy was defined as a pregnancy diagnosed initially by biochemical means at 17 days after ET (serum βhCG level of .100 IU) with consequent evidence of a gestational sac with or without a fetal heart observed on transvaginal ultrasound 28 days after ET).  52 women newly referred to for IVF who had not yet commenced treatment.  97 women were also evaluated with a mean age of 32.7 years (range, 21–44 years) who were attending the Recurrent Miscarriage Clinic and had had at least three sequential first-trimester clinical pregnancy losses.  The fertile control group consisted of 106 women who had at least one child born at term without any major pregnancy complications and without any period of subfertility (i.e.6 months of trying) before conception. These women ranged in age from 19–45 years (mean age 33.6 years).  IVF indications were isolated male factor infertility (35%); occlusive  tubal disease diagnosed by laparoscopy or radiologic examination (27%); unexplained infertility (22%); significant endometriosis  involving the ovaries (8%); combined tubal disease and male factor infertility (8%). |
| **Interventions** | ELISA assay was used to measure immunoglobulin (Ig) G and IgM isotypes of each of anticardiolipin antibody, antiphosphatidylserine, antiphosphatidylethanolamine antiphosphatidylinositol and anti-b2 glycoprotein I antibodies.(The phospholipids obtainded from Sigma (Sydney, New South Wales, Australia) and ELISA plate from Corning High Binding, Corning, Corning, NY). Lupus anticoagulant was determined by using a dilute Russell's viper venom time-based assay.  Statistical analysis included chi-square and Fisher’s exact tests for differences between groups, and multiple linear regression analysis and Spearman’s nonparametric tests for relations between results.  Samples were considered to be positive when the optical density of a sample exceeded the multiple of the median of the 95th percentile of 284 normal serum samples. |
| **Outcomes** | Overall, 84 (23%) of the 360 samples tested positive for at least one autoantibody. 30/105 of the study population were positive to at least one aPL whereas 16/106 of the control group were positive.  Anti-b2 glycoprotein I IgM antibody and antinuclear antibody were significantly associated with both IVF implantation failure and recurrent miscarriage. |

**Bellver 2008**

Jose´ Bellver, Sergio R. Soares, Claudio A lvarez, Elkin Munoz, Alberto Ramirez,Carmen Rubio, Vicente Serra, Jose Remohi, Antonio Pellicer**,** The role of thrombophilia and thyroid autoimmunity in unexplained infertility, implantation failure

and recurrent spontaneous abortion. Human Reproduction Vol.23, No.2 pp. 278–284, 2008

| **Methods** | A prospective study which includes 31 unexplained infertility (UI), 26 implantation failure (IF), 30 women with recurrent abortion compared with 32 controls. |
| --- | --- |
| **Participants** | Women were enrolled between first March, 2004 and first January, 2007. The experimental group consists of 31 women with UI more than one year, 26 with IF at least twice with embryo transfer of at least two good quality embryos, 30 women with recurrent abortions (between 2–9 previous spontaneous abortions). All were younger than 38 years old, with absence of autoimmune or endocrine disorders, with normal ovarian function, normal hysterosalpingography and transvaginal ultrasound scan of the uterus and ovaries, normal karyotype, and whose partner had normal karyotype and normozoospermia.  The control group included 32 women of 18–35 years old, all Caucasian, with normal karyotype, no history of spontaneous abortions, autoimmune disorders or endocrine diseases, and previous term pregnancies without complications. |
| **Interventions** | ELSA assay was used to measure immunoglobulin (Ig) M and IgG anticardiolipin antibodies (QUANTA LITE^TM^ INOVA Diagnostics, Inc., San Diego, USA) ((units for IgM: mPL/ml  and for IgG: gPL/ml ranges: negative ,15, indeterminate 15–20 and positive>20). Lupus anticoagulant was performed using a clotting assay with LA1 screening reagent/LA2 confirmation reagent result was expressed as positive or negative. |
| **Outcomes** | Positive aCL IgG: 0 controls , 3 UI, 1 IF, 0 RSA (non-statistical significant)  Positive aCL IgM: 6 controls, 3 UI, 1 IF, 6 RSA (non-statistical significant). Positive Lupus anticoagulant: 0 controls, 0UI, 3 IF, 1 RSA  (p= 0.056) |
| **Notes** | Women included in the control group were significantly younger and had more live births than in the other three groups. |

**Qublan 2006**

Hussein S.Qublan, Suhair S.Eid, Hani A.Ababneh, Zouhair O.Amarin, Aiman Z.Smadi,Farakaid F.Al-Khafaji, Yousef S.Khader. Acquired and inherited thrombophilia: implication in recurrent IVF and embryo transfer failure.

Human Reproduction Vol.21, No.10 pp. 2694–2698, 2006

| **Methods** | The study group comprised of 90 consecutive women with three or more previously failed IVF–embryo transfer cycles (group A) compared with two control groups: group B (n = 90) women who have had successful pregnancy after their first IVF–embryo transfer cycle, and group C (n = 100) women who conceived spontaneously with at least one uneventful pregnancy and no previous history of miscarriage. |
| --- | --- |
| **Participants** | 90 consecutive women with a history of at least three previously failed IVF–ET treatments, presenting to the infertility clinic between January 2001 and August 2005, were included in this study (group A). Women’s age ranged from 23 to 44 years (mean ± SD,31 ± 4.2).  Women’s age of group B ranged from 22 to 40 years (mean ± SD, 30 ± 3.1) and group C ranged from 17 to 41 years (mean ± SD, 30 ± 2.8). Women with endometriosis, hydrosalpinx, abnormal uterine cavity on the hysterosalpingogram and history of thromboembolic disease and those  who were receiving hormonal treatment were not included in the  study group.  In the IVF/ET cycles, only cycles in which grade 1 and 2 embryos were transferred were included in the study group. Indications for IVF treatment included anovulation, unexplained infertility and male and tubal factor. |
| **Interventions** | Immunoglobulin M (IgM) and IgG anti-cardiolipin were assayed using validated ELISA assay calibrated against international standards. ACL antibodies were reported in international units (positive when >10 IU/ml). LA was performed by the kaolin cephalin clotting time utilizing sensitive reagents and by the dilute Russell’s viper venom time with a neutralization procedure using frozen–thawed platelets. Results for LA were expressed as positive or negative. |
| **Outcomes** | aCL antibodies and LA antibodies were more common in women with  repeated IVF failures (18.9%) compared with 4.4 and 5% in either of the control groups (not statistically significant) .LA:8/90 of group A, 2/90 of group B and 2/100 of group C. ACl 9/90 of group A, 2/90 of group B and 3/100 of group C. |

**E.****Geva 1995**

E.Geva, A.Amit, L.Lerner-Geva, F.Azem, I.Yovel and J.B.Lessing, Autoimmune disorders: another possible cause for in-vitro fertilization and embryo transfer failure. Human Reproduction vol.10 no. 10 pp.256O-2563, 1995

| **Methods** | 50 infertile patients with 3 or more IVF failures versus 40 patients who had conceived and delivered following =/< 3 IVF and embryo |
| --- | --- |
| **Participants** | The study group comprised 50 IVF patients with three or more previously failed cycles after embryo transfer. The study group consisted of  two subgroups according to the cause of infertility: 24 patients were  diagnosed as infertile from mechanical factors [tubal occlusion  proven by hysterosalpingography, no evidence of endometriosis in  laparoscopy and normal postcoital test (PCT) prior to treatment], and  26 as unexplained infertility (unexplained infertility was defined as  normal history and examination of both partners, a history of at least  2 years of infertility, regular ovulation, normal sperm analysis, normal  PCT, normal uterus, patent tubes, no pelvic disease and no sperm  antibodies). The control group comprised 80 computer-matched women: 40 who had conceived and delivered following three or less IVF and embryo transfer cycles and were matched for age, duration and type of infertility  cycles, and 40 who were healthy nulligravidas (*the second control group will not be used in the metanalysis)* |
| **Interventions** | Immunoglobulin IgG anti-cardiolipin detected by ELISA assay (Reads Medical Products, Westminster, CO,USA). Lupus anticoagulant (LA) estimated by the clotting time of citrated plasma was measured by a coagulometer MLA 1000 (Medical Laboratory Automation Inc., Pleasantville, NY, USA). Serum values of ACA <23 GPL IU (IgG phospholipid unit)/ml was considered normal. |
| **Outcomes** | 6.0% (3/50) of patients were positive for anti-cardiolipin antibodies and none of the control group. LA was negative in both study and control groups. |

**Birkenfeld 1994**

A. Birkenfeld, T. Mukaida, L Minichiello, M. Jackson, N.G. Kase,M. Yemeni. Incidence of Autoimmune Antibodies in Failed Embryo Transfer Cycles. Birkenfeld A,

Mukaida T, Minichiello L, Jackson M, Kase NC, Yemini M. AJRI 1994; 31 :65-68

© Munksgaard

| **Methods** | Three groups were studied: group I: 56 patients who failed to conceive following embryo transfer, group II: 14 patients who have conceived following IVF-ET and delivered or are carrying an uncomplicated ongoing pregnancy and group III: 69 patients who were new candidates for IVF-ET. Statistical analysis was performed using Fisher's exact test and Chi-square analysis (two-tailed). |
| --- | --- |
| **Participants** | Participants included in group I, (56 consecutive patients) failed to conceive following one or more embryo transfers (either fresh or frozen)  between October 1992 to September 1993 were studied  for the presence of autoimmune factors. In group II (14 patients) conceived following IVF-ET during the same period and delivered or are carrying an uncomplicated ongoing pregnancy beyond the 12^th^ gestational week, were studied as the control group. In group III 69 patients were new candidates for IVF-ET programs between February and July 1993 were prospectively studied for the presence of autoimmune factors.  The indication for IVF in all patients was tubal mechanical factors.  Couples with male factors were excluded from this study. |
| **Interventions** | Lupus anticoagulant was detected by modified Russell ViperVenom assay through demonstration of prolonged coagulation  times .The presence of IgG and IgM anticardilipin antibodies were detected using ELISA assay. (Reads Medical Products, Inc., Westminster, CO, performed by Roche Biomedical Laboratories) |
| **Outcomes** | 18/56 (32.1 %) of patients in group I tested positive for one or more of the autoimmune antibodies. 0/14 of group II tested positive for autoimmune antibodies *(P<.02).* 7/69 (10%) of group III were found positive to one or more of the autoimmune factor. This rate is significantly lower than the rate of positive autoimmune antibodies detected in group I *(P<.003).*  10/18 from group I were positive for anti-cardiolipin antibodies and 10/18 of group I positve for lupus anticoagulant. |

| **Notes** | Fifteen patients of the 18 who tested positive for  autoimmune antibodies (lupus anticoagulant/ anti-cardiolipin antibodies/ANA) and who had previously failed to conceive following ET underwent a subsequent IVF-ET cycle while being treated with prednisone and aspirin.(10 mg of prednisone daily and 80 mg of aspirin daily) |
| --- | --- |

**Kaider 1996**

Kaider BD, Price DE, Roussev RG, Coulam CB. Antiphospholipid antibody prevalence in patients with IVF failure. AJRI 1996; 35:388-393

| **Methods** | Group from 42 women with IVF failure compared with 42 women who successfully conceived after IVF were tested for the presence of anti-phospholipid antibodies(aPL). |
| --- | --- |
| **Participants** | Participants have had at least 12 embryos transferred during several in vitro fertilization cycles without ensuing pregnancy. Successful post-IVF pregnancy was determined by obtaining two consecutive rising beta-hCG levels followed by an ultrasound to confirm a viable conceptus. |
| **Interventions** | Three isotypes of antibody: IgA, IgG, and IgM against seven phospholipids: cardiolipin , phosphatidylethanolamine , phosphatidylinositol, phosphatidic acid , phosphatidyl-glycerol, phosphatidylcholine and phosphatidyl-serine were tested by ELISA assay (Sigma, St. Louis, MO). The borderline levels were established based  on the 95th percentile and 2.0 multiple of the median.  Positivity was based on the 99th percentile and 2.0 multiple of the median. |
| **Outcomes** | IVF failure group 11/42 (26.2%) were positive for aPLs, the control group, 2/42 (4.8%) were positive only for IgA against phosphatidylethanolamine (*p=0,01)* |

**Buckingham 2006**

K.L.Buckingham, P.R.Stone, J.F.Smith, L.W.Chamley. Antiphospholipid antibodies in serum and follicular fluid is there a correlation with IVF implantation failure? Human Reproduction Vol.21, No.3 pp. 728–734, 2006

| **Methods** | Total of 99 women undergoing IVF, 28 without fertilization success and 71 with fertilization success. |
| --- | --- |
| **Participants** | All women undergoing IVF treatment at Fertility Plus, National Women’s Hospital, Auckland were eligible for entry. Fertilization was determined by  the presence of two adjacent pronuclei 16–18 h later. Embryos were maintained in culture and transferred to the uterus transcervically under ultrasound guidance using a Sydney IVF transfer catheter, 48–72 h after oocyte retrieval. A maximum of three embryos were transferred (mean two embryos) at one time. Fresh embryo transfers occurred in 87 women , 6 women had a ‘freeze all’, whereby all the embryos were cryopreserved. A further 6 women did not proceed to embryo replacement Those women who did not have an embryo replacement were all aPL negative. |
| **Interventions** | Serum was screened for the presence of IgG and IgM class antibodies reactive with β2 glycoprotein I, cardiolipin or phosphatidylserine by ELISA (Biorad Benchmark ELISA plate reader) assay.  When the optical density of a sample exceeded the 99th percentile  of 292 normal serum samples was considered to be positive. |
| **Outcomes** | Women with aPLs had a lower implantation rate (14%) than women without these antibodies (24.1%).Five patients were aPL positive of 22 unsuccessful IVF and 13 patients were aPL positive of 71 fertilization success group. |

**Saxtorph 2020**

Malene Hviid Saxtorph RBMO VOLUME 00 ISSUE 0 2020

| **Methods** | Prospective controlled cohort study. In total, 86 women with a history of reccurent implantation failure compared to controls. |
| --- | --- |
| **Participants** | Between April 2017 and September 2019, 86 women with a history of having undergone at least three consecutive high-quality fresh or frozen embryo transfers after IVF treatment were recruited to the study. Inclusion criteria were failure of implantation after at least three consecutive high-quality embryo transfers in fresh or frozen cycles, a normal uterus and uterine cavity on ultrasound examination, including absence of adenomyosis, non-smoker and age younger than 40 years at referral. Women presenting with a history of endometriosis or signs of endometriosis was not excluded from the RIF group. High-quality embryos were defined as either day-5 blastocysts of at least 4BB according to Gardner's classification or day-2 embryos of at least 4c2b according to the Istanbul consensus criteria for cleavage stage embryos.  A parallel cohort of 37 controls with no history of RIF or previous fertility treatment was also recruited to the study. Screening of possible eligible controls was based on referral diagnosis and a questionnaire on gynaecological history. |
| **Interventions** | Beta-2 glycoprotein IgG and IgM, were measured using Dimension Vista® 1500 and Centaur XP (Siemens, Munich, Germany), anti-cardiolipin IgG and IgM were measured using ImmunoCAP 250 (Thermo Fisher), lupus anti-coagulant was measured using Sysmex CS-5100 (Siemens). All analyses were carried out at the Department of Clinical Biochemistry, Zealand University Hospital, and are validated and accredited (DS/EN ISO 15189). |
| **Outcomes** | The peripheral blood samples were subject to measurements of antiphospholipid antibodies (lupus anti-coagulant, anti-cardiolipin immunoglobulin G [IgG] and immunoglobulin M [IgM], beta-2-glycoprotein IgG and IgM |

**Appendix 3: Risk of Bias**

**NEWCASTLE - OTTAWA QUALITY ASSESSMENT SCALE**

**
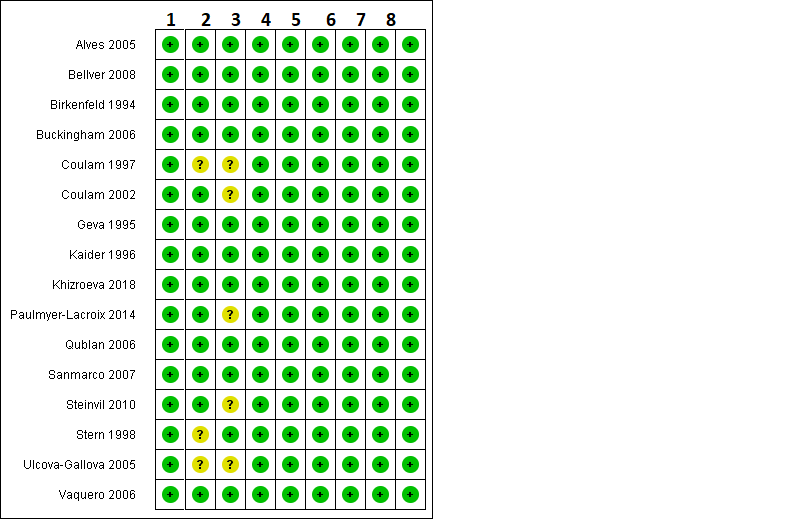
**

1. Adequate definition of the case.
2. Representativeness of the cases
3. Selection of Controls
4. Definition of Controls
5. Comparability of cases and controls on the basis of the design analysis
6. Ascertainment of exposure
7. Same method of ascertainment for cases and controls
8. Non-Response rate
